# Cardiotrophin‐1 therapy reduces disease severity in a murine model of glomerular disease

**DOI:** 10.14814/phy2.16129

**Published:** 2024-07-02

**Authors:** Nuria Perretta‐Tejedor, Karen L. Price, Daniyal J. Jafree, Gideon Pomeranz, Maria Kolatsi‐Joannou, Carlos Martínez‐Salgado, David A. Long, Elisavet Vasilopoulou

**Affiliations:** ^1^ Developmental Biology and Cancer Research and Teaching Department UCL Great Ormond Street Institute of Child Health London UK; ^2^ UCL Centre for Kidney and Bladder Health London UK; ^3^ Department of Physiology and Pharmacology, Translational Research on Renal and Cardiovascular Diseases (TRECARD) University of Salamanca, Institute of Biomedical Research of Salamanca (IBSAL) Salamanca Spain; ^4^ Specialised Foundation Programme in Research NHS East of England Cambridge UK; ^5^ Comparative Biomedical Sciences The Royal Veterinary College London UK

**Keywords:** cardiotrophin‐1, fibrosis, glomerulus, inflammation

## Abstract

Cardiotrophin‐1 (CT‐1), a member of the interleukin (IL)‐6 cytokine family, has renoprotective effects in mouse models of acute kidney disease and tubulointerstitial fibrosis, but its role in glomerular disease is unknown. To address this, we used the mouse model of nephrotoxic nephritis to test the hypothesis that CT‐1 also has a protective role in immune‐mediated glomerular disease. Using immunohistochemistry and analysis of single‐cell RNA‐sequencing data of isolated glomeruli, we demonstrate that CT‐1 is expressed in the glomerulus in male mice, predominantly in parietal epithelial cells and is downregulated in mice with nephrotoxic nephritis. Furthermore, analysis of data from patients revealed that human glomerular disease is also associated with reduced glomerular CT‐1 transcript levels. In male mice with nephrotoxic nephritis and established proteinuria, administration of CT‐1 resulted in reduced albuminuria, prevented podocyte loss, and sustained plasma creatinine, compared with mice administered saline. CT‐1 treatment also reduced fibrosis in the kidney cortex, peri‐glomerular macrophage accumulation and the kidney levels of the pro‐inflammatory mediator complement component 5a. In conclusion, CT‐1 intervention therapy delays the progression of glomerular disease in mice by preserving kidney function and inhibiting renal inflammation and fibrosis.

## 1 INTRODUCTION

Glomerular disease occurs due to impairment of the kidney filtration barrier consisting of endothelial cells, the glomerular basement membrane and podocytes. Disruption of any of these components can result in defective filtration leading to proteinuria and is a major cause of progression to end stage kidney disease (ESKD) (Benzing & Salant, 2021). Glomerular disease is often accompanied by an inflammatory response characterized by secretion of chemokines, infiltration of leukocytes and subsequent fibrosis which correlates with declining renal function (Kurts et al., 2013; Zoja et al., 2006). Therefore, therapies that modulate the inflammatory response have the potential to improve kidney function and significantly reduce the morbidity and mortality associated with glomerular disease.

Cardiotrophin‐1 (CT‐1) is a member of the interleukin‐6 (IL‐6) family of cytokines that signals through a receptor complex comprised of glycoprotein 130 (gp130) and leukaemia inhibitory factor receptor (LIFR) (Lopez‐Yoldi et al., 2015; Pennica et al., 1995). CT‐1 has protective effects in the context of heart, nervous system and liver disease (Lopez‐Yoldi et al., 2015). In contrast, administration of CT‐1 in healthy rats over a prolonged period results in cardiac, vascular and renal fibrosis (Lopez‐Andres et al., 2012) highlighting the importance of maintaining optimum levels of CT‐1 to preserve organ function.

With regard to kidney disease, CT‐1 administration is protective in rat models of acute kidney disease induced by either gentamicin (Quiros et al., 2016), contrast media (Quiros et al., 2013) or ischemia–reperfusion (Garcia‐Cenador et al., 2013, 2018) resulting in improved renal function and lower levels of pro‐inflammatory cytokines and leukocyte‐adhesion molecules. In a mouse model of tubulointerstitial fibrosis induced by unilateral ureteral obstruction, lack of endogenous CT‐1 exacerbates tubular injury and fibrosis resulting in enhanced renal expression of adhesion molecules and macrophage markers indicating increased inflammation. Additionally, CT‐1 treatment of either CT‐1 knockout or wild‐type mice improves tubular injury, inflammation, and fibrosis in this model, highlighting a protective role for CT‐1 in tubulointerstitial injury (Perretta‐Tejedor et al., 2019).

Podocytes express gp130 (Nagayama et al., 2014), indicating that they can respond to CT‐1. However, the role of CT‐1 in the setting of glomerular injury is unknown. We hypothesized that akin to its role in tubulointerstitial injury, CT‐1 may also be protective in glomerular disease by mediating inflammation and fibrosis. We subsequently show that glomerular disease induced by nephrotoxic serum (NTS) is associated with reduced expression of CT‐1 in parietal epithelial cells. Additionally, therapeutic intervention with CT‐1 reduces levels of albuminuria, inflammation and fibrosis in mice with established glomerular disease induced by NTS. Thus, we provide evidence that CT‐1 treatment can improve the progression of established glomerular disease in mice.

## 2 METHODS

### 2.1 Single‐cell RNA sequencing analysis

The raw single‐cell RNA sequencing (scRNAseq) dataset used in this analysis was acquired from a study characterising the single‐cell transcriptome of glomerular disease models using the 10X Genomics platform (Chung et al., 2020). Matrices of gene counts per droplet, generated after alignment of reads to genes, were acquired from the National Center for Biotechnology Information Gene Expression Omnibus (GSE146912) and are available at https://www.ncbi.nlm.nih.gov/geo/query/acc.cgi?acc=GSE146912. scRNAseq analysis was performed as previously described (Mason et al., 2022) using RStudio for Macintosh (RStudio Inc., v1.2.5042). Single‐cell transcriptome data derived from nephrotoxic nephritis samples obtained 1 (*n =* 2) and 5 (*n =* 2) days after injection with NTS and controls (*n* = 2) were analyzed and the FindAllMarkers function used to compare the scaled expression of *Ctf1*, the gene encoding CT‐1, between datasets. In the cell clusters where *Ctf1* transcripts were detected, the average log fold change (log_2_FC) was calculated between experimental conditions. Wilcoxon rank sum tests were used to assess statistical significance. The R script use for analysis has been made publicly available at the following GitHub repository: https://github.com/daniyal‐jafree1995/collaborations/blob/main/PerettaTejedoretal_v3.R.

### 2.2 Nephroseq analysis

We used Nephroseq version 5 and analyzed a dataset (Ju et al., 2013) to compare glomerular *CTF1* transcript levels in patients and healthy living donors. In this dataset, gene transcript profiling of micro‐dissected glomerular samples from patients with lupus nephritis, vasculitis, IgA nephropathy, membranous glomerulonephropathy, minimal change disease, focal segmental glomerulosclerosis, arterial hypertension and diabetic nephropathy and living donors was analyzed on Affymetrix Human U133 Plus 2.0 and Affymetrix Human U133A (altCDF v10) platforms (Ju et al., 2013).

### 2.3 Experimental animals and procedures

All experiments were carried out in accordance with the UK Animals (Scientific Procedures) Act 1986 and the ARRIVE guidelines with both Home Office and institutional ethical approval (University College London Local Ethics Committee). Mice were fed Teklad global 18% protein diet (2018, Inotiv, Huntington, UK). Male C57BL/6 mice aged between 7 and 10 weeks were preimmunized by subcutaneous injection of sheep immunoglobulin G (IgG) (250 μg, I5131, Sigma‐Aldrich) in complete Freund's adjuvant (F5881, Sigma‐Aldrich), followed by intravenous administration of sheep NTS (250 μL) 5 days later. The NTS was a kind gift from Professor Michael Robson (King's College London, UK) (Brown et al., 2006; Vasilopoulou et al., 2016). To assess the therapeutic effect of CT‐1, mice with glomerular injury were injected intravenously with either CT‐1 (400 μg/kg body weight, *n* = 8, 250‐25, ≥98% purity, Peprotech, Cranbury, NJ, USA) or saline (*n* = 9) at day 9, 13 and 16 following NTS administration; a dose regime chosen based on our previous studies demonstrating a protective effect of CT‐1 administration in renal injury (Garcia‐Cenador et al., 2013; Perretta‐Tejedor et al., 2019). Mice were humanely euthanised 21 days following NTS administration by exposure to carbon dioxide in a rising concentration followed by dislocation of the neck.

### 2.4 Renal function

Urine was collected from mice by housing them individually in metabolic cages overnight. Blood samples were collected from the lateral saphenous vein. Albumin concentrations were measured by enzyme‐linked immunosorbent assay (Dessapt‐Baradez et al., 2014; Long et al., 2013) (E90‐134, Bethyl Laboratories, Montgomery, TX). Plasma creatinine concentrations were measured using isotope dilution electrospray mass spectrometry (Greenberg et al., 2012; Vasilopoulou et al., 2016). A commercially available kit was used to measure blood urea nitrogen (BUN) (DIUR‐100, BioAssay Systems, Hayward, CA) (Kolatsi‐Joannou et al., 2011).

### 2.5 Tissue processing and immunostaining

Tissues were fixed in 4% paraformaldehyde in PBS. Following tissue dehydration and paraffin embedding, 5 μm‐thick sections were cut and stained with periodic acid‐Schiff (PAS) reagent (395132 and 3952016, Sigma‐Aldrich) or Sirius red (365548, Sigma‐Aldrich). PAS staining was assessed by two blinded observers as described previously (Vasilopoulou et al., 2016) and each glomerulus was assigned one of the following scores; 0, normal glomerular structure; 1, increased mesangial matrix deposition and hypercellularity with some loss of capillary loops; 2, increased matrix deposition and focal areas of sclerosis; 3, >50% of glomerulus sclerotic with very few capillary loops; 4, >75% of glomerulus sclerotic and presence of glomerular epithelial hyperplasia lesions. Fifty glomeruli were assessed per mouse and the average score was calculated for each mouse. Sirius red staining was quantified in a minimum of 10 fields of view per mouse to calculate the fibrotic area in glomeruli and the surrounding tubulointerstitium using Image Pro Plus (Media Cybernetics, Bethesda, MD) (Perretta‐Tejedor et al., 2019). Immunohistochemistry was performed using antibodies against CT‐1 (MAB438, R&D systems) or F4/80 (MCA497R, AbD Serotec, Oxford, UK) followed by secondary rabbit anti‐rat antibody (BA‐4001, Vector Laboratories, Burlingame, CA) and ImmPRESS polymer anti‐rabbit IgG reagent (MP7401, Vector Laboratories) conjugated to horseradish peroxidase and detected by 3,3′‐diaminobenzidine (D4293, Sigma‐Aldrich). Glomerular CT‐1 immunostaining was scored by two blinded observers and designated as either weak (score = 0), moderate (score = 1) or strong (score = 2). Fifty glomeruli were assessed per mouse and the results are presented as the average score per mouse. F4/80+ cells were counted in at least 10 glomeruli per sample within the glomerular tuft and in the peri‐glomerular region (Vasilopoulou et al., 2016). Immunofluorescence was performed using an antibody against Wilms Tumor 1 (WT1) (AP15857PU‐S, Acris Antibodies, Herford, Germany) followed by anti‐rabbit AlexaFluor594 secondary antibody (A21207, Thermo Fisher Scientific). The number of WT1+ cells within each glomerular tuft were counted and the glomerular tuft area was measured using ImageJ (Schneider et al., 2012) in 15 glomeruli per mouse. Data are presented as the mean number of WT1+ cells per glomerular tuft and as the number of WT1+ cells per μm^2^ of glomerular tuft area. Negative controls consisted of omission of primary antibodies.

### 2.6 Quantitative real‐time PCR

RNA was extracted from mouse whole kidney. 500 ng was used to prepare cDNA (iScript kit, Bio‐Rad, Hemel Hempstead, UK) and qRT‐PCR performed as described previously (Long et al., 2013) for *Ctf1*, the gene encoding CT‐1 using a commercially available assay (qMmuCID0020747, Bio‐Rad). The following primers were used for *Hprt* as a housekeeping gene; forward primer: 5′‐AAGCTTGCTGGTGAAAAGGA‐3′ and reverse primer: 5′‐GCAAATCAAAAGTCTGGGGA‐3′. All measurements were performed in duplicate.

### 2.7 Cytokine arrays

Cytokine levels were assessed in whole‐kidney lysates (300 μg) using the mouse cytokine array (ARY006, R&D Systems) according to the manufacturer's instructions. Four samples were assessed per group. Quantification was carried out using ImageJ (Schindelin et al., 2012) and the data were normalized to the background reading on the corresponding membrane and presented as fold change in cytokine levels compared to the control group.

### 2.8 Statistical analysis

All samples were assessed by independent observers blinded to treatment group. Data are presented as mean ± SD and were analyzed using GraphPad Prism v9 (GraphPad Software, La Jolla, CA). Normal distribution was assessed by Shapiro–Wilk test. For comparisons of two groups, data were analyzed using unpaired *t* test. When three or more groups were assessed, one‐way ANOVA with Holm–Sidak multiple comparison post hoc tests was used. Data affected by two variables were analyzed using two‐way ANOVA with Holm–Sidak multiple comparison post hoc tests. When data were not normally distributed, it was analyzed by Kruskal–Wallis non‐parametric test followed by Dunn post hoc tests. Statistical significance was accepted at *p* ≤ 0.05.

## 3 RESULTS

### 3.1 CT‐1 is downregulated in a murine model of glomerulonephritis and in human glomerular disease

**FIGURE 1:**
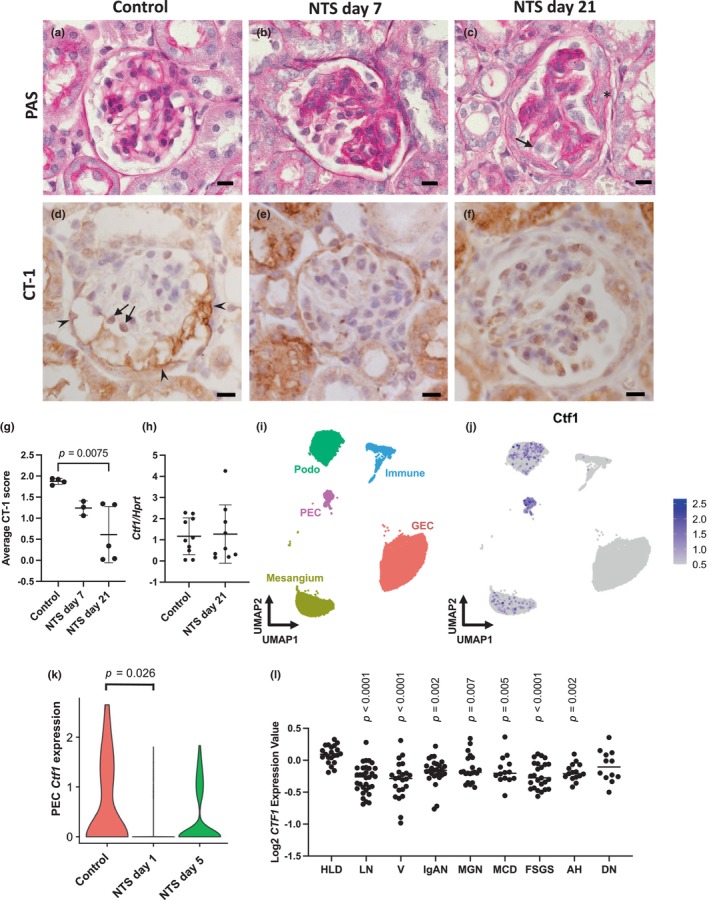
CT‐1 downregulation in the glomeruli of mice with NTS nephritis. (a–c) Representative images of glomerular morphology assessed by PAS and haematoxylin staining in healthy mice and NTS‐treated mice 7 and 21 days post‐NTS administration. The arrow indicates adhesion between the glomerular tuft and the Bowman's capsule, * indicates an epithelial hyperplasia lesion. (d–f) Representative images showing immunostaining for CT‐1 in healthy mice and NTS‐treated mice 7 and 21 days post‐NTS administration. Arrows indicate CT‐1 immunostaining within the glomerular tuft and arrowheads indicate CT‐1 immunostaining in the Bowman's capsule. Scale bars = 10 μm. (g) Quantification of CT‐1 immunostaining in glomeruli from healthy mice and NTS‐treated mice 7 and 21 days post‐NTS administration. Each glomerulus was assigned a score of 0, 1 and 2. Control, *n* = 4; NTS day 7, *n* = 3; NTS day 21, *n* = 5. Data are presented as the mean ± SD and analyzed by one‐way ANOVA with Holm‐Sidak post hoc test. (h) Quantification of *Ctf1* mRNA levels in whole‐kidney homogenates of healthy control (*n* = 10) and NTS‐treated mice 21 days post‐NTS administration (*n* = 9). Data are presented as the mean ± SD and analyzed by unpaired *t* test. (i) Uniform manifold approximation and projection (UMAP) of a scRNAseq dataset (GSE146912) containing glomerular cells from healthy (control) mice, and those 1 or 5 days post‐NTS administration. (j) Feature plot demonstrating *Ctf1* transcript expression across cell types in the dataset, demonstrating enrichment in PECs. Scaled expression of *Ctf1* was clipped to a minimum value of 0.5. (k) Violin plot comparing the scaled expression of *Ctf1* in parietal epithelial cells between control (*n =* 38 cells) and NTS‐treated mice one (*n =* 270 cells, log_2_FC = 0.80 compared to control, *p* = 0.026) and 5 days (*n =* 386 cells, log_2_FC = 0.70 compared to control, *p* = 0.071) post‐NTS administration. (l) Comparison of *CTF1* mRNA levels in microdissected glomerular samples from CKD patients (lupus nephritis, LN, *n* = 32; vasculitis, V, *n* = 23; IgA nephropathy, IgAN, *n* = 27; membranous glomerulonephropathy, MGN, *n* = 21; minimal change disease, MCD, *n* = 14; focal segmental glomerulosclerosis, FSGS, *n* = 25; arterial hypertension, AH, *n* = 15; diabetic nephropathy, DN, *n* = 12) and healthy living donors (HLD, *n* = 21). Data are presented as individual data points and the median and analyzed by Kruskal–Wallis with Dunn's multiple comparisons to the control group (HLD). GEC, glomerular endothelial cell, PEC, parietal epithelial cell.

To induce glomerular disease, we utilized the accelerated NTS nephritis model, where mice are pre‐immunised by subcutaneous injection of sheep immunoglobulin, followed by intravenous administration of NTS (Papakrivopoulou et al., 2018; Vasilopoulou et al., 2016). This protocol results in irreversible and progressive glomerular injury, demonstrated by glomerulosclerosis and tubulointerstitial fibrosis accompanied by leukocyte infiltration, replicating some of the pathologic features seen in human crescentic glomerulonephritis (Foster, 2016). We collected kidney samples 7 (*n* = 3) and 21 (*n* = 5) days after NTS induction and compared CT‐1 glomerular expression patterns in the early and late phases of the NTS nephritis model to those in healthy mice (*n* = 4). In healthy control mice (Figure 1a), CT‐1 protein was localized in cells within the glomerular tuft and strongly in the parietal layer of the Bowman's capsule and in the Bowman's space (Figure 1d). Seven days after NTS administration, there was mild glomerular injury, featuring increased mesangial matrix (Figure 1b) with a concurrent reduction in CT‐1 immunoreactivity (Figure 1e). The reduction in CT‐1 was more pronounced 21 days after NTS administration when glomerular injury was more severe and involved collapse of capillary loops, glomerulosclerosis, adhesions between the glomerular tuft and the Bowman's capsule and glomerular epithelial hyperplasia lesions (Figure 1c) and CT‐1 expression in the glomerular tuft and the parietal epithelium was diminished (Figure 1f). This was confirmed by semi‐quantitative analysis of the glomerular CT‐1 protein levels performed by two blinded observers, who examined 50 glomeruli per mouse and scored the degree of staining in each glomerulus as either 0 (weak or none), 1 (moderate) or 2 (strong) (Figure S1). Healthy control mice had an average score of 1.88 ± 0.07. NTS mice had a lower score of 1.24 ± 0.17 at 7 days and a significantly (*p* = 0.008) reduced score of 0.61 ± 0.67 at 21 days compared with healthy mice (Figure 1g). The mRNA levels of *Ctf1*, the gene encoding CT‐1, were similar in whole‐kidney lysates from healthy control and NTS mice at 21 days (Figure 1h), indicating that CT‐1 is specifically downregulated in the glomerulus in the NTS model.

To further interrogate the expression of CT‐1 in the glomerulus, we analyzed publicly available transcriptomic data. We used a published scRNAseq dataset obtained from glomeruli of healthy or NTS‐injured mice (Figure 1i) (Chung et al., 2020). Transcripts for *Ctf1*, the gene encoding CT‐1, were predominantly enriched within parietal epithelial cells as compared to other glomerular cell types of healthy and injured mice, with scant expression by podocytes and mesangial cells (Figure 1j). We then compared parietal epithelial cell *Ctf1* transcript levels in healthy and NTS‐injured mice. There was a statistically significant reduction (log_2_ fold change = 0.80) in parietal epithelial cell *Ctf1* transcript levels 1 day post‐NTS administration compared with controls (*p* = 0.026). Five days post‐NTS administration, there was a log_2_ fold change of 0.70 in parietal epithelial cell *Ctf1* transcript levels compared with controls (*p* = 0.071) (Figure 1k).

To assess whether the glomerular level of CT‐1 is also altered in human diseases, we analyzed a dataset (Ju et al., 2013) available on the Nephroseq platform to compare the levels of *CTF1* in microdissected glomeruli from patients with glomerular disease and healthy living donors (Figure 1l). There was a significant reduction in *CTF1* mRNA levels in patients with lupus nephritis (*p* < 0.0001), vasculitis (*p* < 0.0001), IgA nephropathy (*p* = 0.002), membranous glomerulonephropathy (*p* = 0.007), minimal change disease (*p* = 0.005), focal segmental glomerulosclerosis (*p* < 0.0001) and arterial hypertension (*p* = 0.002), but not in patients with diabetic nephropathy (*p* = 0.139), indicating that CT‐1 signaling is downregulated in a range of human glomerular diseases.

### 3.2 CT‐1 intervention therapy improves albuminuria in murine glomerulonephritis

**FIGURE 2:**
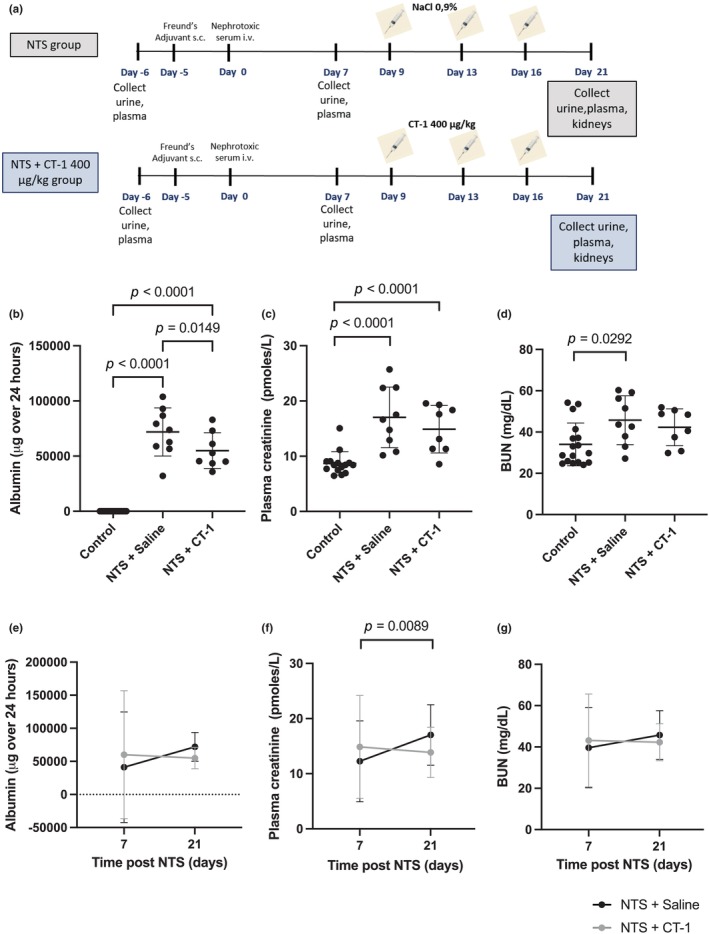
Assessment of renal function following NTS injury and treatment with CT‐1. (a) Outline of experimental strategy. (b) Twenty‐four hour urinary albumin excretion (c) plasma creatinine and (d) blood urea nitrogen concentration (BUN) before the induction of glomerular disease (control group) and 21 days after NTS injection (NTS + saline and NTS + CT‐1; one‐way ANOVA with Holm‐Sidak post hoc test). (e) Twenty‐four hour urinary albumin excretion (f) plasma creatinine and (g) BUN concentration 7 and 21 days after NTS injection (two‐way repeated measures ANOVA with Holm–Sidak post hoc test). Control, *n* = 17; NTS + Saline, *n* = 9; NTS + CT‐1, *n* = 8. Data are presented as the mean ± SD.

We subsequently hypothesized that since CT‐1 was downregulated in NTS nephritis and CT‐1 treatment has been shown to improve other models of kidney disease induced by unilateral ureteral obstruction (Perretta‐Tejedor et al., 2019), ischemia (Garcia‐Cenador et al., 2013) and toxins (Quiros et al., 2016), it may also have a protective effect in glomerulonephritis. To test this, we used an intervention therapeutic strategy (Figure 2a), akin to the approach likely to be required if CT‐1 therapy was used for patients with kidney disease. NTS nephritis was induced in 17 mice and 7 days after NTS administration, overnight urine was collected in metabolic cages to assess albuminuria as a marker of glomerular injury (Agrawal & Smoyer, 2017). Animals were then grouped based on their albuminuria measurements and randomly assigned to be injected either saline (NTS + saline, *n* = 9) or CT‐1 (400 mg/kg, NTS + CT‐1, *n* = 8) intravenously at day 9, 13 and 16 following NTS administration. 7 days after NTS injection, there was no significant difference in the 24‐h albumin excretion levels, plasma creatinine or BUN between the groups of NTS nephritis mice to be administered either saline or CT‐1 (Figure S2).

Nephritis mice administered either saline or CT‐1 were evaluated 21 days after NTS injection. At 21 days, albumin excretion over 24 h was significantly increased in NTS mice administered saline versus levels before disease induction (*p* < 0.0001). In nephropathic mice administered CT‐1, albumin excretion was significantly lower compared with those administered saline (*p* = 0.015) (Figure 2b). Plasma creatinine was significantly increased in nephropathic mice treated with either saline or CT‐1 compared with levels before disease induction (*p* < 0.0001, Figure 2c). BUN was significantly increased in nephropathic mice treated with saline (*p* = 0.029) but not in those treated with CT‐1, compared with levels before disease induction (Figure 2d). We also measured albumin excretion, plasma creatinine and BUN longitudinally between 7 and 21 days in each of the nephropathic mice. Overall, renal function worsened in the mice administered saline, conversely mice administered CT‐1 did not show further deterioration of renal function. Mean albuminuria levels in nephropathic mice administered saline were 41,108 ± 8353 μg/24 h at day 7 and 71,875 ± 2183 at day 21 (*p* = 0.536). In contrast, in nephropathic mice administered CT‐1 albuminuria levels were 60,062 ± 9662 at day 7 and 54,964 ± 1620 at day 21 (*p* = 0.874; Figure 2e). Plasma creatinine levels in NTS nephritis mice administered saline increased from 12.25 ± 7.33 pmoles/L at day 7 to 17.03 ± 5.49 at day 21 (*p* = 0.009). In contrast, nephropathic mice administered CT‐1 had similar plasma creatinine levels at day 7 (14.86 ± 9.33) and day 21 (14.89 ± 4.29) (*p* = 0.530; Figure 2f). BUN levels were 39.62 ± 19.40 mg/dL at day 7 and 45.74 ± 11.85 at day 21 in NTS mice administered saline (*p* = 0.188). In NTS mice administered CT‐1, BUN levels were 43.16 ± 22.49 at day 7 and 42.30 ± 8.91 at day 21 (*p* = 0.325; Figure 2g). Assessment of gross glomerular morphology in NTS mice revealed a range of abnormalities including collapse of capillary loops, segmental or global glomerulosclerosis, adhesion of the glomerular tuft to the Bowman capsule, and glomerular epithelial hyperplasia lesions and a significantly increased mean glomerular score compared to healthy mice (*p* < 0.0001), but there was no significant difference between NTS mice administered saline or CT‐1 (*p* = 0.836; Figure S3).

### 3.3 CT‐1 intervention therapy protects the integrity of the glomerular filtration barrier

**FIGURE 3:**
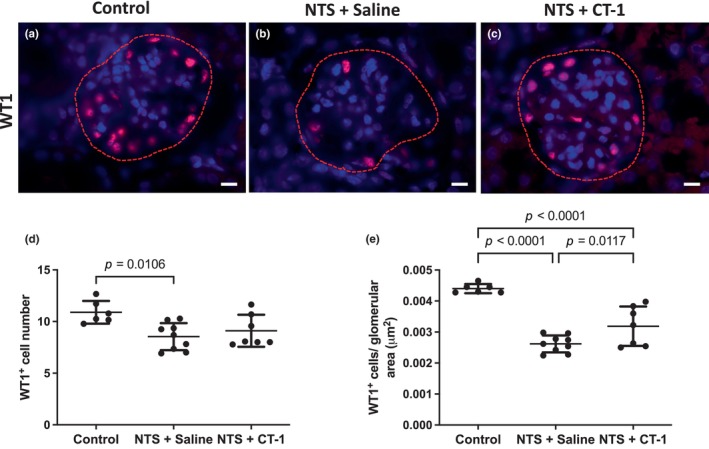
Podocyte assessment following NTS injury and treatment with CT‐1. Representative images of glomeruli from control (a), NTS + Saline (b) and NTS + CT‐1 (c) treated mice stained for WT1. Dashed line indicates glomerular tuft boundary. Scale bar = 10 μm. Quantification of (d) the mean number of WT1+ cells in the glomerular tuft and (e) the number of WT1+ cells in the glomerular tuft normalized to the glomerular tuft area 21 days after NTS injection. Individual data points represent average values per mouse and 15 glomeruli were assessed per mouse. Healthy controls, *n* = 6; NTS + Saline, *n* = 9; NTS + CT‐1, *n* = 7. Data are presented as the mean ± SD and analyzed by one‐way ANOVA with Holm–Sidak post hoc test.

As previous studies have indicated that mouse podocytes express the CT‐1 co‐receptor gp130, (Nagayama et al., 2014) we hypothesized that CT‐1 administration might prevent podocyte loss in nephrotoxic nephritis. We therefore quantified the number and density of WT1+ podocytes in our model (Figure 3a–c). The number of podocytes (mean ± SEM) in the glomerular tuft of nephropathic mice (8.54 ± 1.31) was significantly reduced compared to healthy mice (10.89 ± 1.10; *p* = 0.011). In CT‐1 treated mice the number of podocytes (9.11 ± 1.55) was not significantly different to healthy (*p* = 0.054) or nephropathic (*p* = 0.410) mice (Figure 3d). Nephrotoxic nephritis also resulted in reduced podocyte density (number of WT1+ cells normalized to the area of the glomerular tuft) in nephropathic mice treated with saline (*p* < 0.0001) or CT‐1 (*p* < 0.0001) compared with healthy controls. CT‐1 treatment of nephropathic mice significantly increased podocyte density compared with saline (*p* = 0.012; Figure 3e).

### 3.4 CT‐1 intervention therapy improves fibrosis in murine glomerulonephritis

**FIGURE 4:**
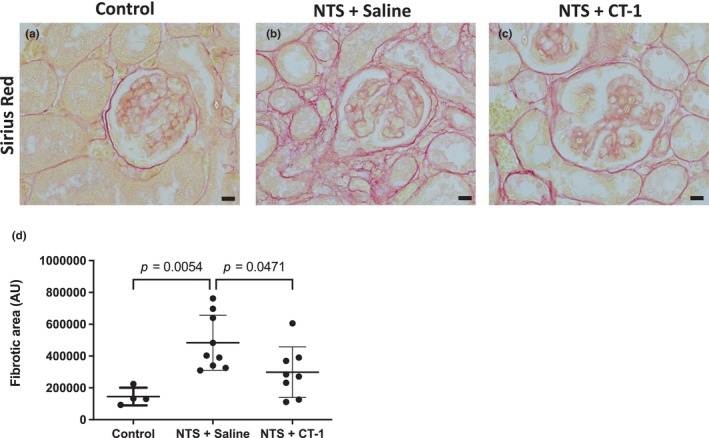
Assessment of fibrosis following NTS injury and treatment with CT‐1. Representative images of the cortical area of kidneys from control (a), NTS + Saline (b) and NTS + CT‐1 (c) treated mice stained with Sirius Red 21 days after NTS injection. Scale bar = 10 μm. (d) Quantification of the Sirius red‐positive area (fibrotic area). Individual data points represent average values per mouse and a minimum of 10 images were assessed per mouse. Healthy controls, *n* = 4; NTS + Saline, *n* = 9; NTS + CT‐1, *n* = 8. Data are presented as the mean ± SD and analyzed by one‐way ANOVA with Holm‐Sidak post hoc test.

Glomerulosclerosis and interstitial fibrosis are common features of disease progression in NTS nephritis (Meng et al., 2014). Since CT‐1 improves interstitial fibrosis after unilateral ureteral obstruction in mice (Perretta‐Tejedor et al., 2019), we sought to determine if it has anti‐fibrotic effects in NTS nephritis. Renal fibrosis was assessed by Sirius Red staining to visualize collagen fibres and quantitative analysis was performed focusing on the glomeruli and the surrounding tubulointerstitium (Figure 4a–c). The fibrotic area was significantly increased (*p* = 0.005) in nephropathic mice compared with healthy controls. Treatment with CT‐1 significantly reduced the fibrotic area in nephropathic mice compared with saline administration (*p* = 0.047, Figure 4d).

### 3.5 CT‐1 intervention therapy reduces macrophage accumulation and renal cytokine expression

**FIGURE 5:**
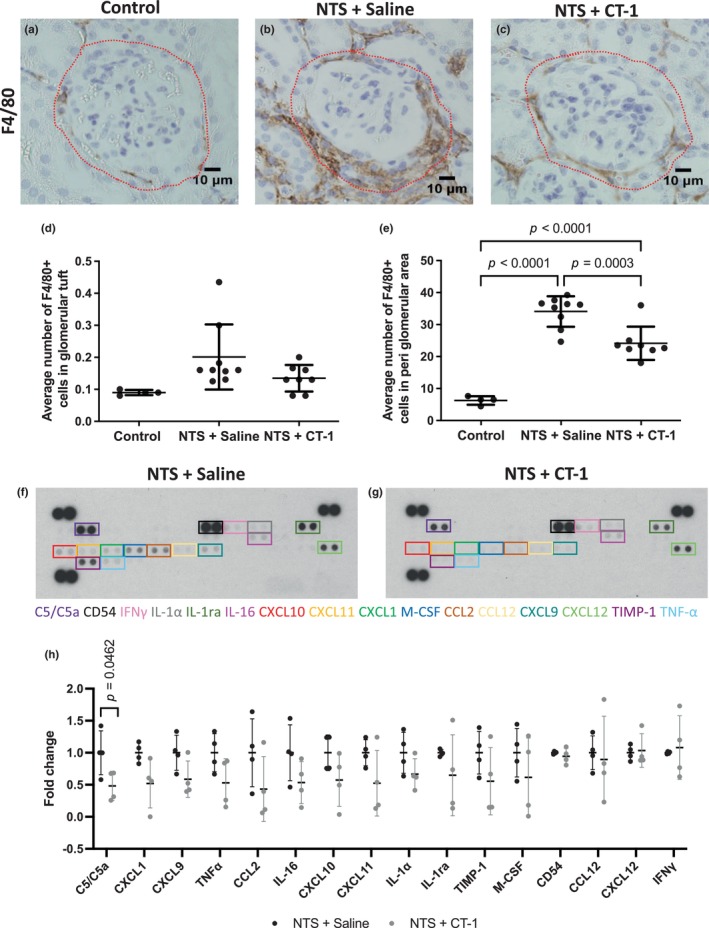
Assessment of inflammation following NTS injury and treatment with CT‐1. Representative images of glomeruli from control (a), NTS + Saline (b) and NTS + CT‐1 (c) treated mice stained for F4/80 21 days after NTS injection. Scale bar = 10 μm. Quantification of (d) the number of F4/80+ cells in the glomerular tuft and (e) the number of F4/80+ cells in the peri‐glomerular area (indicated by the dotted line). Individual data points represent average values per mouse and a minimum of 10 glomeruli were assessed per mouse. Healthy controls, *n* = 4; NTS + Saline, *n* = 9; NTS + CT‐1, *n* = 8. Data are presented as the mean ± SD and analyzed by one‐way ANOVA with Holm–Sidak post hoc test. Representative images of immunoblots showing detection of cytokines and chemokines in kidney lysates from NTS + Saline (f) and NTS + CT‐1 (g) mice. (h) Quantification of the fold‐change in cytokine/chemokine levels between NTS + Saline (*n* = 4) and NTS + CT‐1 (*n* = 4) kidneys. Data are presented as the mean ± SD and analyzed by unpaired *t* test.

Infiltration of immune cells, particularly macrophages, plays a critical role in the progression of NTS nephritis and the establishment of fibrosis (Duffield et al., 2005). Indeed, we found significant macrophage accumulation assessed by immunostaining for F4/80 in mice with NTS nephritis (Figure 5a–c). We subsequently quantified the number of F4/80+ cells in the glomerular tuft (Figure 5d) and the surrounding peri‐glomerular region (Figure 5e, see dotted line) and found that there was a significant increase in the number of F4/80+ cells in the peri‐glomerular region of nephropathic mice administered saline (34.1 ± 4.8) compared with healthy controls (6.3 ± 1.3, *p* < 0.0001). Treatment with CT‐1 significantly reduced the number of F4/80+ cells (24.1 ± 5.2, *p* = 0.0003) compared with nephropathic mice treated with saline.

Finally, to identify potential molecular mechanisms by which CT‐1 reduces macrophage accumulation in the injured kidney, we assessed the levels of 40 cytokines, chemokines and acute phase proteins in kidney lysates from nephropathic mice treated with saline or with CT‐1 (Figure 5f,g). Sixteen of these cytokines were detected and there was an overall trend of reduced cytokine levels in CT‐1 treated compared with saline treated mice (12 out of 16 cytokines), with a significant decrease in complement component 5a (C5/C5a) (*p* = 0.046; Figure 5h), a complement molecule that promotes renal inflammation and fibrosis (Boor et al., 2007; Zhang et al., 2020).

## 4 DISCUSSION

Glomerular disease involves both injury of glomerular cells and inflammatory responses that drive disease progression. It has long been recognized that the IL‐6 family of cytokines contribute to inflammatory glomerular disease (Fukatsu et al., 1991; Malide et al., 1995; Morel et al., 2000). In this study, we focused on the role of CT‐1, a member of the IL‐6 cytokine family that had not been examined previously in the context of glomerular disease. We have shown that CT‐1 is enriched in parietal epithelial cells within the glomerulus. Furthermore, in murine NTS nephritis and across a range of glomerular diseases in humans, we observe a reduction of glomerular CT‐1 expression. Administration of CT‐1 to mice with established glomerular disease improves proteinuria, preserves plasma creatinine levels and ameliorates inflammation and fibrosis. Thus, we provide evidence that treatment with CT‐1 can halt disease progression in experimental glomerulonephritis.

It has been previously reported that CT‐1 expression is localized to tubular epithelial cells and is increased in the unilateral ureteral obstruction (UUO) mouse model that involves tubulointerstitial fibrosis (Perretta‐Tejedor et al., 2019). However, the expression of CT‐1 in the glomerulus and how that varies with glomerular disease had not been previously explored. Here, we have demonstrated expression of CT‐1 protein in the mouse glomerulus, in the glomerular tuft and predominantly in the parietal epithelium. We further show reduction of CT‐1 expression in the parietal epithelium following the induction of NTS nephritis in mice, demonstrated by immunohistochemistry. Analysis of a scRNAseq dataset (Chung et al., 2020) demonstrated reduction of CT‐1 mRNA levels specifically in parietal epithelial cells 1 day post‐NTS administration in mice. Although there are differences in the timepoints assessed, the sources of the NTS and the protocol utilized in the present study and the work by Chung et al. (2020), the fact that CT‐1 downregulation was observed in both studies strengthens the evidence that this is a feature of NTS nephritis. Importantly, analysis of data from microdissected glomeruli from patients with chronic kidney disease (CKD) and healthy living donors (Ju et al., 2013) showed reduced glomerular levels of CT‐1 in a range of glomerular diseases, revealing that CT‐1 signalling is also disrupted in human glomerular disease. Determining the protein expression of CT‐1 in human kidney biopsies by immunohistochemistry would add important information about the expression patterns of CT‐1 in human glomerular disease.

We further demonstrated that enhancing CT‐1 signalling is beneficial in experimental glomerulonephritis. Administration of CT‐1 to NTS‐injected mice with severe albuminuria halted glomerular disease progression as evidenced by reduced albuminuria and sustained plasma creatinine levels and podocyte density in CT‐1 compared with saline‐treated nephropathic mice 2 weeks after the initiation of CT‐1 treatment. It should be noted that our experiments were conducted using male mice and further work is needed to confirm if the results are applicable to female mice. CT‐1 exerts its action by binding to the gp130 and LIFR receptor complex (Lopez‐Yoldi et al., 2015; Pennica et al., 1995). The protein expression of gp130 has been demonstrated in the mouse glomerulus and localized predominantly to podocyte cells, identified by synaptopodin, with weak expression in the endothelium and mesangium (Nagayama et al., 2014). LIFR expression in the glomerulus has not been characterized in detail, however, LIF signalling induces nephron development (Barasch et al., 1999) and LIFR knockout mouse embryos have reduced kidney size and fewer comma shaped bodies and premature glomeruli indicating impaired nephrogenesis (Kosfeld et al., 2017). It is therefore possible that podocytes have the potential to respond to CT‐1 signalling. In support of this, we found that, alongside the reduction of albuminuria, treatment with CT‐1 maintained podocyte density in the glomerular tuft of nephropathic mice. CT‐1 may therefore improve albuminuria by binding on its receptor on podocyte cells to promote their survival or adhesion to the glomerular basement membrane. Previous studies have shown that CT‐1 promotes the survival of cardiac myocytes (Liao et al., 2002) and neurons (Bordet et al., 1999; Oppenheim et al., 2001). However, podocyte‐specific deletion of gp130 did not affect glomerular function or histopathology in healthy mice or mice injured with NTS thus questioning the importance of gp130 signalling in podocytes, albeit podocyte number was not assessed in that study (Nagayama et al., 2014). Further work using alternative models of glomerular injury could reveal whether CT‐1 administration is beneficial in other types of glomerular disease.

In the tubulointerstitium, treatment with CT‐1 reduced NTS‐induced fibrosis and macrophage accumulation, consistent with previous studies demonstrating anti‐fibrotic and anti‐inflammatory effects of CT‐1 in the UUO mouse model (Perretta‐Tejedor et al., 2019) and in a rat model of ischaemia‐reperfusion injury (Garcia‐Cenador et al., 2013). This effect could be mediated by glomerular‐tubular crosstalk (Rana et al., 2024). Alternatively, CT‐1 may act directly on renal tubular epithelial cells and fibroblasts which express gp130 and LIFR, (Perretta‐Tejedor et al., 2019) to inhibit pro‐inflammatory and pro‐fibrotic responses. We identified that CT‐1 treatment reduced the levels of the complement component, C5a, in the kidney. C5a is generated during complement activation and is a potent inflammatory mediator that promotes the release of inflammatory cytokines and leukocyte chemotaxis. (Ricklin et al., 2010) In the kidney, C5a binds on its receptors, C5aR1 and C5aR2, on myeloid cells, tubular epithelial cells and fibroblasts and promotes inflammation and fibrosis. (Boor et al., 2007; Li et al., 2017; Peng et al., 2019; Zhang et al., 2020) C5a signalling promotes disease progression in rodent experimental models of tubulointerstitial fibrosis, (Boor et al., 2007) acute and chronic pyelonephritis, (Choudhry et al., 2016; Li et al., 2017) renal ischaemia‐reperfusion injury, (Peng et al., 2019) and immune complex glomerulonephritis (Alexander et al., 2012). It is therefore likely that the anti‐inflammatory and anti‐fibrotic effects of CT‐1 are at least partially mediated via downregulation of C5a.

A limitation of our study is that we have not examined the downstream signalling mechanisms that mediate the beneficial effect of CT‐1 in this model. CT‐1 binding to the LIFR leads to heterodimerisation with gp130 and activation of the Janus‐activated kinase (JAK)/signal transducer and activator of transcription (STAT) signaling pathway (Jones et al., 2011; Lopez‐Yoldi et al., 2015). However, STAT activation is a feature of several experimental models of kidney disease and is thought to contribute to renal fibrosis and disease progression, (Bienaime et al., 2016; Chuang & He, 2010; Dai et al., 2013; Zheng et al., 2019) therefore, it is unlikely that its activation by CT‐1 accounts for the beneficial effect observed in our study. The pro‐survival effect of CT‐1 is mediated by alternative pathways including mitogen‐activated protein kinase (MAPK), phosphatidylinositol 3‐kinase (PI3K)/Akt and nuclear factor κB (NFκB) signaling (Lopez‐Yoldi et al., 2015), and some of these pathways may be responsible for the beneficial effects of CT‐1 observed in our study.

In conclusion, our study demonstrates that CT‐1 can maintain glomerular function and delay disease progression in mice with established glomerular disease. Together with previous studies demonstrating a renoprotective effect of CT‐1 in the context of acute (Garcia‐Cenador et al., 2013, 2018; Quiros et al., 2013, 2016) and chronic (Perretta‐Tejedor et al., 2019) kidney injury, CT‐1 is emerging as a promising therapeutic target in kidney disease.

## FUNDING INFORMATION

This work was supported by a KRUK Postdoctoral Fellowship (PDF8/ 2015 to EV), project grants from the Medical Research Council (MR/P018629/1 and MR/J003638/1) and a Wellcome Trust Investigator Award (220895/Z/20/Z) to DAL, a Diabetes UK studentship (17/0005733 to DAL). DJ was supported by a Foulkes Foundation postdoctoral fellowship and the Specialised Foundation Programme in the East of England Foundation Schools. NPT was supported by an ERA‐EDTA Short Term fellowship and PhD fellowship from “Junta de Castilla y León and the European Social Fund”, Spain (EDU/346/2013). Professor Long's laboratory is supported by the NIHR Biomedical Research Centre at Great Ormond Street Hospital for Children NHS Foundation Trust and University College London.

## CONFLICT OF INTEREST STATEMENT

The authors have no conflicts of interest.

## ETHICS STATEMENT

All experiments were carried out in accordance with the UK Animals (Scientific Procedures) Act 1986 and the ARRIVE guidelines with both Home Office and institutional ethical approval (University College London Local Ethics Committee).

## Supporting information


Figure S1.

Figure S2.

Figure S3.

## Data Availability

The datasets generated and/or analyzed during the current study are available from the corresponding author on reasonable request.
