# Supplementary figures and images for: Cardiotrophin‐1 therapy reduces disease severity in a murine model of glomerular disease
Source: Physiol Rep. 2024 Jul 2;12(13):e16129. doi: 10.14814/phy2.16129 (PMC11219243; doi:10.14814/phy2.16129)

# Supplementary figure 1

Score 0

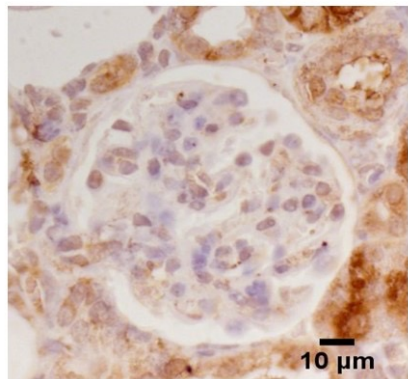

Score 1

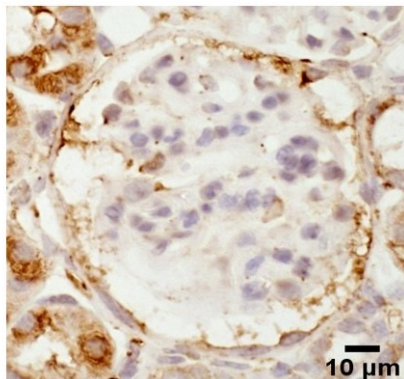

Score 2

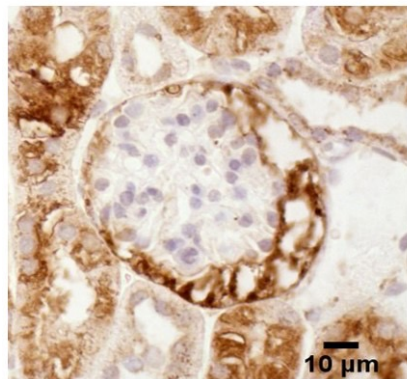

Supplement: Supplementary file 1 — Figure S1. [file PHY2-12-e16129-s003.pdf]

## Supplementary figure 2

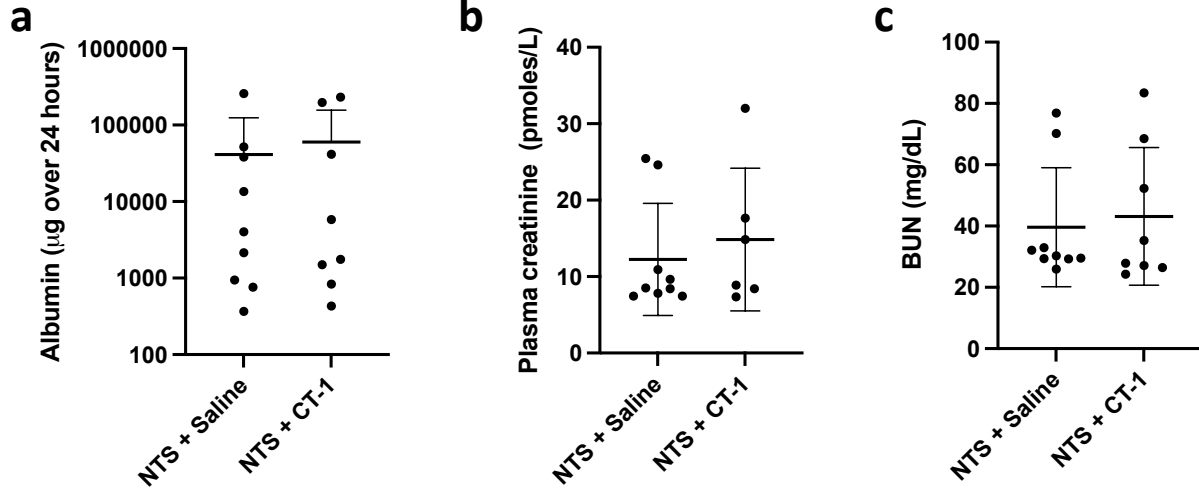

Supplement: Supplementary file 2 — Figure S2. [file PHY2-12-e16129-s002.pdf]

# Supplementary Figure 3

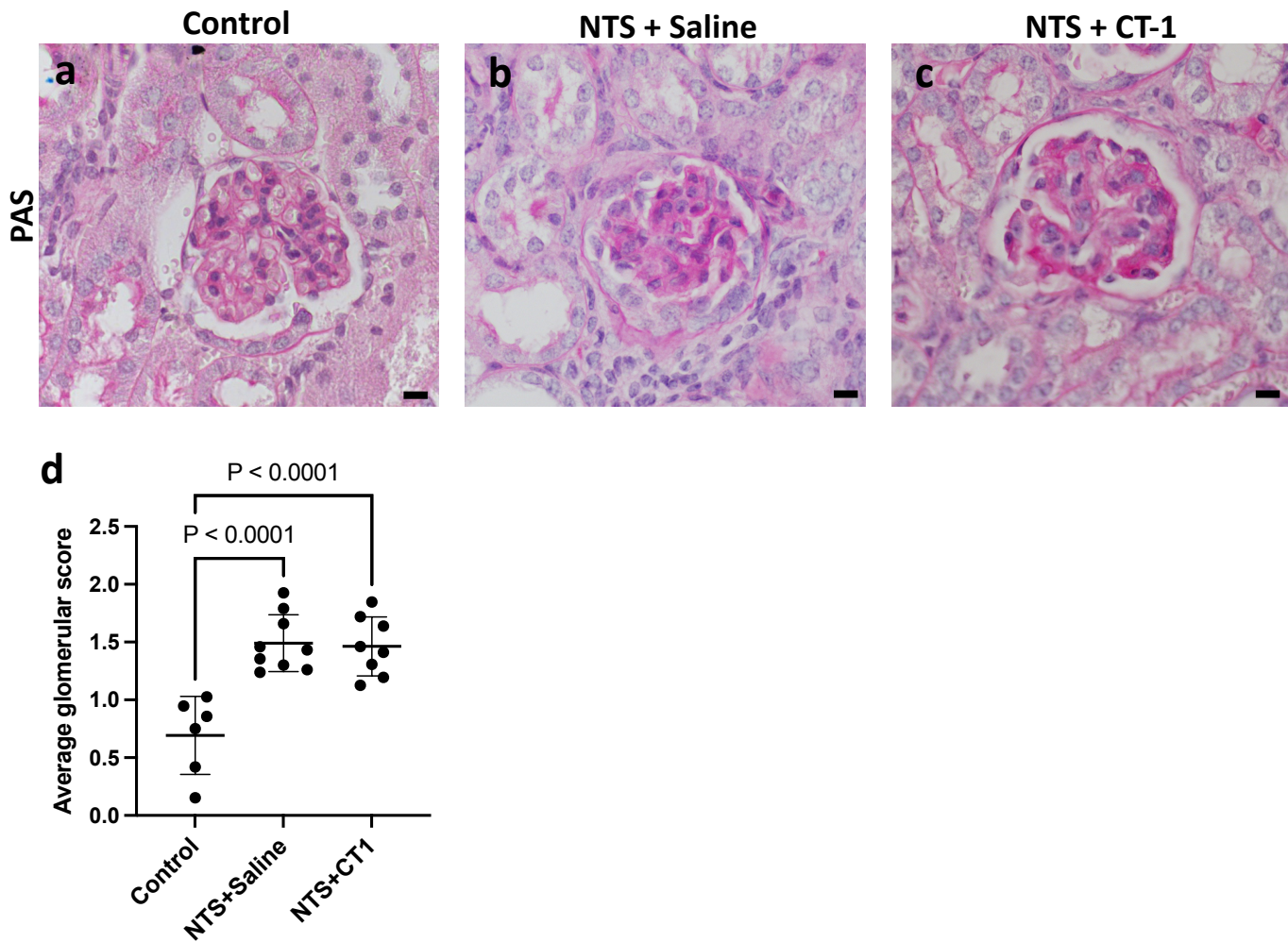

Supplement: Supplementary file 3 — Figure S3. [file PHY2-12-e16129-s001.pdf]
